# Supplementary material for: Vitamin E (300 mg) in the treatment of MASH: A multi-center, randomized, double-blind, placebo-controlled study
Source: Cell Rep Med. 2025 Feb 18;6(2):101939. doi: 10.1016/j.xcrm.2025.101939 (PMC11866479; doi:10.1016/j.xcrm.2025.101939)
Supplement: Document S1. Figures S1–S4 and Tables S1–S5 [file mmc1.pdf]

Cell Reports Medicine, Volume *6*

Supplemental information

Vitamin E (300 mg) in the treatment of MASH:

A multi-center, randomized, double-blind,

placebo-controlled study

Yu Song, Wenjing Ni, Minghua Zheng, Huiping Sheng, Jing Wang, Shilong Xie, YongFeng Yang, Xiaoling Chi, Jinjun Chen, Fangping He, Xiaotang Fan, Yuqiang Mi, Jing Zhang, Bingyuan Wang, Lang Bai, Wen Xie, Bihui Zhong, Yee Hui Yeo, Fajuan Rui, Shufei Zang, Jie Li, Junping Shi, and the Chinese NAFLD Clinical Research Network (CNAFLD CRN)

Supplementary Information

Table of Content

Supplementary Figures

Figure S1. Information collection of diet and exercise. Related to STAR Methods.

Figure S2. Typical picture with improvements in histology. Related to Figure 2.

Figure S3. Safe characteristics of the study subjects (Safety set). Related to Table 3.

Figure S4. Blood concentration of vitamin E. Related to STAR Methods.

Supplementary Tables

Table S1 Sensitivity analyses (mITT). Related to Figure 2.

Table S2 Subgroup analyses. Related to Figure 2.

Table S3 Changes in other histologic features in mITT. Related to Figure 3.

Table S4. Change between baseline and week 96 in secondary endpoints and after 120 weeks (96 weeks of treatment followed by 24 weeks off-treatment). Related to Table 2.

Table S5 Combination therapy. Related to Table 3.

Figure S1. Information collection of diet and exercise. Related to STAR Methods.

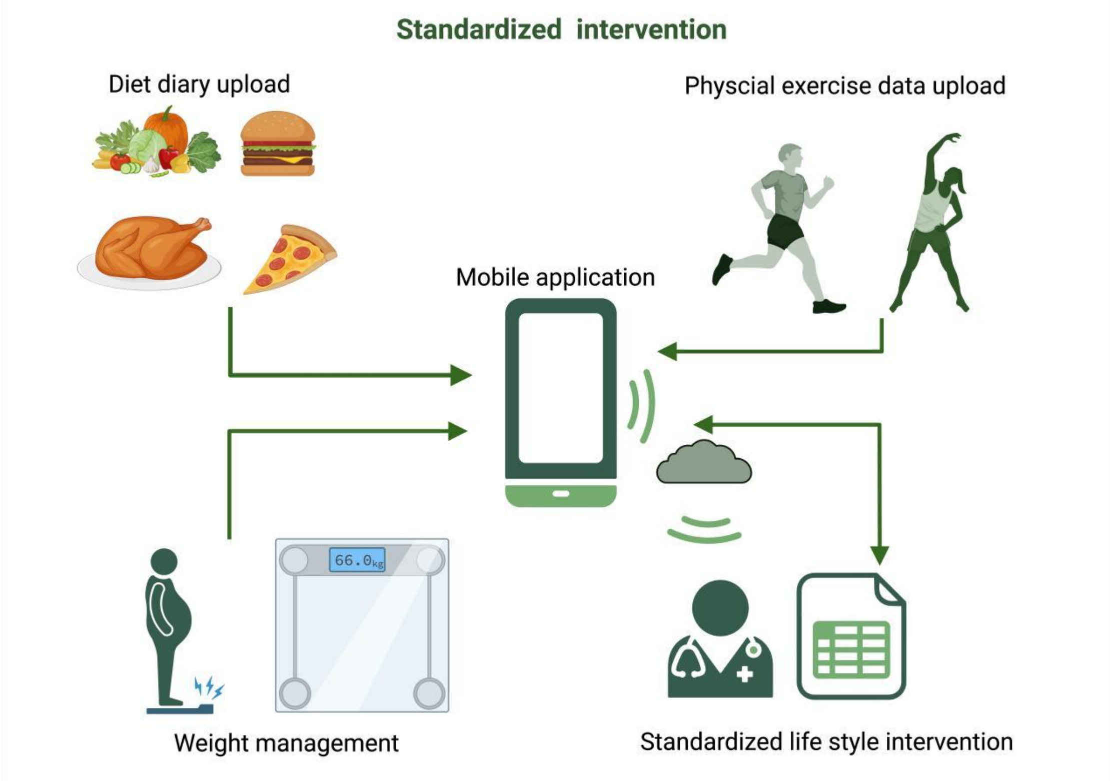

All participants were given personalized lifestyle advice by dieticians. Every patient's daily food consumption and exercise were collected using a mobile phone application.

Figure S2. Typical picture with improvements in histology. Related to Figure 2.

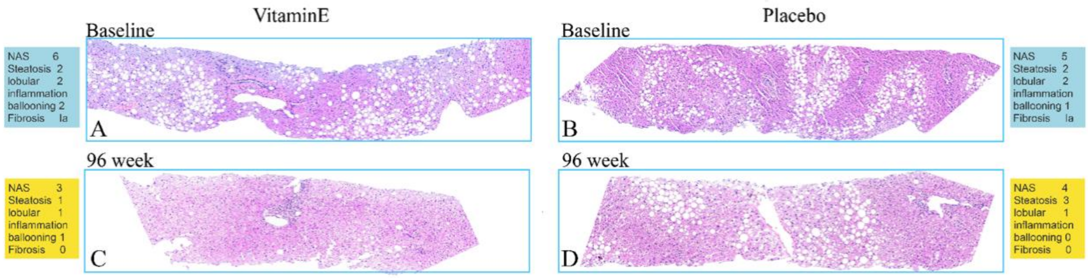

Micrographs of a patient with marked histological improvement after 96 weeks of treatment. Panel A (Vitamin E group H&E) and B (Placebo group H&E) from the pre-treatment biopsy showed the classic features of steatohepatitis. Panel C (Vitamin E group H&E) and D (Placebo group H&E) from the post-treatment biopsy showed the improvement in all features of steatohepatitis. Abbreviations: H&E: hematoxylin-eosin.

Figure S3. Safe characteristics of the study subjects (Safety set). Related to Table 3.

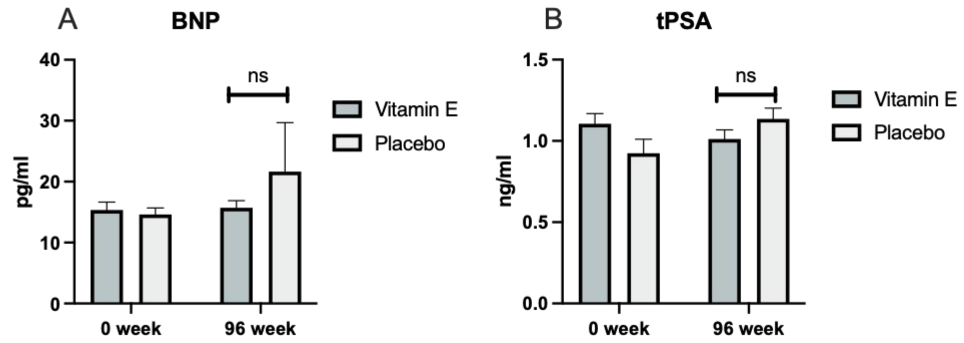

(A): BNP levels are assessed in each group within the safety set population (n=58 in the vitamin E group; n=66 in the placebo group); (B): tPSA levels are assessed in males of each group within the safety set population (n=42 in the vitamin E group; n=50 in the placebo group). Data are presented as mean in each group. Error bars represent standard deviations. Abbreviations: BNP, brain natriuretic peptide; tPSA, total prostate specific antigen.

Figure S4. Blood concentration of vitamin E. Related to STAR Methods.

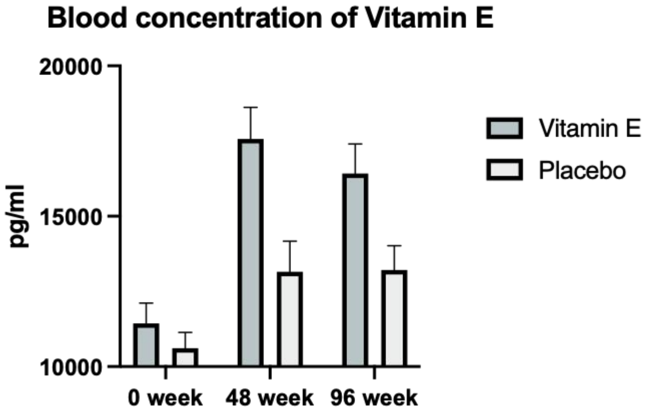

Data are presented as mean in each group. In each time point, the number in the vitamin E and the placebo groups are 39 and 51, respectively. Error bars represent standard deviations.

Table S1. Sensitivity analyses (mITT). Related to Figure 2.

	Vitamin E 300mg Group (n=58)	Placebo group (n=64)	Odds ratio (95% CI)	*P* value
Exclude if missed or outside time window	17 (43.6)	9 (17.6)	3.6 (1.3, 10.7)	0.01
Local reading at baseline, central reading at week 96				
Exclude if missing or outside time window	20 (51.3)	15 (29.4)	1.7 (1.0, 2.9)	0.04
Exploratory secondary endpoints				
Fibrosis improvement	15 (25.9)	10 (15.6)	1.7 (0.8, 3.4)	0.16
Subjects with improvement (%)	25.7%	15.7%		
Sensitivity analyses				
Exclude if missed or outside time window	13 (33.3)	9 (17.6)	2.0 (1.0, 3.9)	0.09
Among subjects F1-3	15 (50.0)	10 (25.6)	2.0 (1.0, 3.7)	0.04
Local reading at baseline, central reading at week 96				
Exclude if missing or outside time window	17 (43.6)	10 (19.6)	2.2 (1.1, 4.3)	0.01
Among subjects F1-3	17 (35.4)	10 (17.2)	2.1 (1.0, 4.1)	0.03

Data are presented with mean (standard deviation).

Table S2. Subgroup analyses for primary outcome (mITT). Related to Figure 2.

	Vitamin E 300mg group (n = 58)	Placebo group (n = 64)	Odds ratio (95% CI)	*P* value	*P* for Interaction
Hp genotype					
Hp 2-1	1 (100.0%)	0 (0.0%)	NA	0.08	0.99
Hp 2-2	16 (28.1%)	9 (14.5%)	(0.8, 6.5)	0.07	
Gender					
Male	14 (33.3%)	5 (10.2%)	4.4 (1.3, 17.1)	0.01	0.06
Female	3 (18.8%)	4 (26.7%)	0.6 (0.08, 4.7)	0.60	
Age at baseline					
< 40	10 (30.3%)	3 (8.1%)	4.9 (1.1, 30.2)	0.02	0.18
≥40	7 (28.0%)	6 (22.2%)	1.4 (0.3, 5.9)	0.63	
BMI					
< 25	4 (22.2%)	5(14.7%)	1.7 (0.3, 9.0)	0.50	0.52
≥25	13 (32.5%)	4 (13.3%)	3.1 (0.8, 14.7)	0.06	
NAS					
2-4	3 (13.0%)	4 (14.3%)	0.9 (0.1, 6.0)	0.90	0.13
5-8	14 (40.0%)	5 (13.9%)	4.1 (1.2, 16.6)	0.01	

Data are presented with n (%). Abbreviations: CI, confidence interval; NAS, Non-alcoholic fatty liver disease score; BMI, body mass index; NA, not applicable.

Table S3. Changes in other histologic features (mITT). Related to Figure 3.

	Vitamin E 300mg group (n = 58)	Placebo group (n = 64)	*P* value
Steatosis			0.13
n (%) worse	2 (3.5%)	8 (12.5%)	
n (%) same	32 (55.2%)	37 (57.8%)	
n (%) better	24 (41.4%)	19 (29.7%)	
Lobular inflammation			0.07
n (%) worse	2 (3.5%)	10 (15.6%)	
n (%) same	35 (60.3%)	31 (48.4%)	
n (%) better	21 (36.2%)	23 (35.9%)	
Hepatocellular ballooning			0.60
n (%) worse	8 (13.8%)	7 (10.9%)	
n (%) same	40 (69.0%)	41 (64.1%)	
n (%) better	10 (17.2%)	16 (25.0%)	
Total NAFLD Activity Score			0.29
n (%) worse	5 (8.6%)	12 (18.8%)	
n (%) same	27 (46.6%)	26 (40.6%)	
n (%) better	26 (44.8%)	26 (40.6%)	
Fibrosis			0.09
n (%) worse	3 (5.2%)	11 (17.2%)	
n (%) same	38 (65.5%)	39 (60.9%)	
n (%) better	17 (29.3%)	14 (21.9%)	

Data are presented with n (%).

Table S4. Change between baseline and week 120 in secondary endpoints (mITT). Related to Table 2.

	Vitamin E 300 mg group (n = 58)	Placebo group (n = 64)	Point estimate (95% CI)	*P*-Value
**Changes in anthropometric parameters**				
BMI, kg/m^2	-0.1 (3.4)	0.1 (1.8)	0.5 (-0.3, 1.3)	0.24
WHR*	0.0 (0.1)	0.0 (0.1)	0.0 (0.0, 0.0)	0.60
**Liver enzyme**				
ALT, U/L	-10.1 (44. 8)	-11.5 (39. 8)	1.5 (-11.2, 14.2)	0.81
AST, U/L	-4.1 (18.6)	-3.4 (17.7)	-2.3 (-7.5, 2.8)	0.38
γ-GT, U/L	0.2 (31.7)	-7.3 (30.3)	6.9 (2.8, 16.6)	0.16
AKP, U/L	-1.1 (31.6)	-4.6 (18.7)	4.1 (-3.8, 12.0)	0.31
TBIL, μmol/L	-1.5 (9.7)	-0.7 (7.7)	-2.0 (-4.0, 0.2)	0.07
**Lipids**				
TG, mmol/L	0.3 (1.2)	0.4 (1.4)	-0.1 (-0.6, 0.3)	0.56
TC, mmol/L	0.4 (1.1)	0.3 (0.9)	0.2 (-0.2, 0.5)	0.28
HDL*, mmol/L	0.1 (0.2)	0.0 (0.2)	0.1 (0.0, 0.2)	0.01
LDL, mmol/L	0.5 (1.1)	0.1 (0.8)	0.4 (0.1, 0.7)	0.01
**Metabolic factors**				
Fasting serum glucose*, mmol/L	0.1 (1.4)	0.3 (1.4)	-0.4 (-0.9, 0.2)	0.17
Fasting insulin, pmol/L	19.2 (104.0)	3.9 (87.0)	15.4 (-13.3, 44.1)	0.29
HOMA-IR	6.8 (34.2)	1.5 (21.1)	5.3 (-3.8, 14.5)	0.25
2h blood glucose, mmol/L	-0.2 (2.0)	0.4 (2.5)	-0.7 (-1.4, 0.0)	0.05
**FibroScan Assessment**				
Controlled attenuation parameter, dB/m	3.0 (66.6)	6.0 (54.2)	-2.8 (-18.2, 12.7)	0.72
Liver stiffness measuremen, kPa	-1.4 (3.3)	1.0 (6.4)	-1.5 (-3.2, 0.2)	0.09

Data are presented with mean (SD). An analysis of covariance (ANCOVA) model was used, with the absolute change from baseline as the dependent variable, treatment group as a fixed factor, and baseline value as a covariate.

* Both baseline BMI and corresponding baseline value were included as covariates in ANCOVA model considering the *P*-value for the main effect of baseline BMI was less than 0.05.

Abbreviations: BMI, body mass index, calculated as weight in kilograms divided by height in meters squared; WHR, waist-to-hip ratio, calculated as waist circumference in meters divided by hip circumference in meters; ALT, alanine aminotransferase; AST, aspartate aminotransferase; γ-GT, Glutamyl peptidyl transferase; AKP, alkaline phosphatase; TG, total triglycerides; TC, total cholesterol; LDL, low-density lipoprotein; HDL, high-density lipoprotein; FPG, fasting plasma glucose; FINS, fasting insulin; HOMA-IR, Homeostasis Model Assessment for Insulin Resistance, calculated as insulin × glucose/22.5, where the unit of measure for insulin is μIU/mL and the unit of measure for glucose is mmol/L; CAP, controlled attenuation parameter; LSM, liver stiffness measurement.

Table S5. Combination therapy (FAS). Related to Table 3.

	Vitamin E (n=58)		Placebo (n=66)	
	n	%	n	%
Cardiovascular system	14	24.1	8	12.5
Calcium channel blockers	5	8.6	3	4.7
Amlodipine	2	3.5	2	3.1
Levoamlodipine Maleate	2	3.5	0	0.0
Felodipine	0	0.00	1	1.6
Nifedipine	1	1.7	0	0.0
Drugs that act on the renin-angiotensin system	4	6.9	4	6.3
Valsartan	0	0.0	3	4.7
Indapamide; Perindopril tert-butylamine	0	0.0	1	1.6
Hydrochlorothiazide; Irbesartan	1	1.7	0	0.0
Hydrochlorothiazide; Valsartan	1	1.7	0	0.0
Telmisartan	1	1.7	0	0.0
Benazepril Hydrochloride	1	1.7	0	0.0
Lipid Regulatory Drugs	1	1.7	2	3.1
Atorvastatin Calcium	1	1.7	1	1.6
Pitavastatin calcium	0	0.0	1	1.6
Rosuvastatin Calcium	0	0.0	1	1.6

Data are presented with n (%).
