# Supplementary material for: Vitamin E (300 mg) in the treatment of MASH: A multi-center, randomized, double-blind, placebo-controlled study
Source: Cell Rep Med. 2025 Feb 18;6(2):101939. doi: 10.1016/j.xcrm.2025.101939 (PMC11866479; doi:10.1016/j.xcrm.2025.101939)
Supplement: Document S2. Article plus supplemental information [file mmc2.pdf]

Cell Reports
Medicine

Article

Vitamin E (300 mg) in the treatment of MASH: A multi-center, randomized, double-blind, placebo-controlled study

Graphical abstract

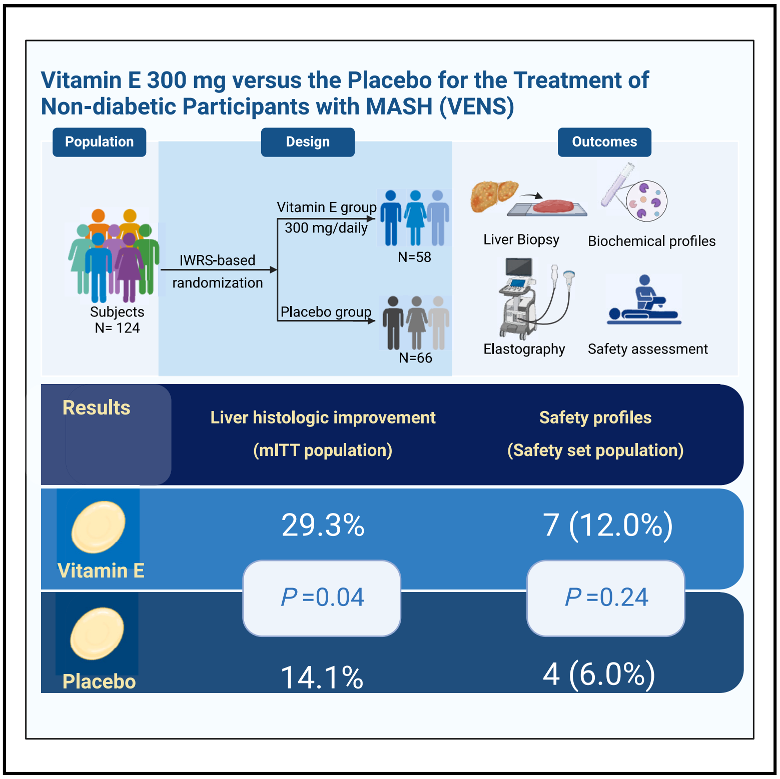

Highlights

- MASH is a leading cause of chronic liver diseases worldwide
- Vitamin E 300 mg improves liver histology and liver functions in MASH patients
- No adverse events are considered to be related to the treatment
- This clinical trial proves the promising future usage of vitamin E

Authors

Yu Song, Wenjing Ni, Minghua Zheng, ..., Jie Li, Junping Shi, the Chinese NAFLD Clinical Research Network (CNAFLD CRN)

Correspondence

lijier@nju.edu.cn (J.L.),
sophiazsf@fudan.edu.cn (S.Z.),
20131004@hznu.edu.cn (J.S.)

In brief

Song et al. prove that vitamin E 300 mg per day for 96 weeks significantly improves liver histology, including steatosis, lobular inflammation, and fibrosis stages; reduces liver stiffness measurement; and decreases liver enzymes and proinflammatory cytokines levels, while it does not show a great effect on lipid and glucose profiles.

Song et al., 2025, Cell Reports Medicine 6, 101939
February 18, 2025 © 2025 The Author(s). Published by Elsevier Inc.
https://doi.org/10.1016/j.xcrm.2025.101939

CellPress

Cell Reports Medicine

CellPress
OPEN ACCESS

Article

Vitamin E (300 mg) in the treatment of MASH: A multi-center, randomized, double-blind, placebo-controlled study

Yu Song,[1,2,21] Wenjing Ni,[3,4,21] Minghua Zheng,[5,21] Huiping Sheng,[6,21] Jing Wang,[7,21] Shilong Xie,[8] YongFeng Yang,[9] Xiaoling Chi,[10] Jinjun Chen,[11] Fangping He,[12] Xiaotang Fan,[12] Yuqiang Mi,[13] Jing Zhang,[14] Bingyuan Wang,[15] Lang Bai,[16] Wen Xie,[17] Bihui Zhong,[18] Yee Hui Yeo,[19] Fajuan Rui,[3,4] Shufei Zang,[20,*] Jie Li,[3,4,*] Junping Shi,[1,2,22,*] and the Chinese NAFLD Clinical Research Network (CNAFLD CRN)

[1]Department of Infectious Diseases and Hepatology, The Affiliated Hospital of Hangzhou Normal University, Hangzhou, Zhejiang 310015, P.R. China
[2]Zhejiang Key Laboratory of Medical Epigenetics, Hangzhou, Zhejiang 310015, P.R. China
[3]Department of Infectious Diseases, Nanjing Drum Tower Hospital Clinical College of Nanjing University of Chinese Medicine, Nanjing, Jiangsu 210000, P.R. China
[4]Department of Infectious Disease, Nanjing Drum Tower Hospital, Affiliated Hospital of Medical School, Nanjing University, Nanjing, Jiangsu 210000, P.R. China
[5]MAFLD Research Center, Department of Hepatology, The First Affiliated Hospital of Wenzhou Medical University, Wenzhou 325015, P.R. China
[6]Department of Infectious Diseases, General Hospital of Ningxia Medical University, Yinchuan, Ningxia 750003, P.R. China
[7]The Affiliated Traditional Chinese Medicine Hospital of Southwest Medical University, Luzhou, Sichuan 646099, P.R. China
[8]Zhejiang Medicine Co. Ltd, Hangzhou, Zhejiang 311899, P.R. China
[9]Department of Liver Disease, the Second Hospital of Nanjing, Affiliated to Nanjing University of Chinese Medicine, Nanjing, Jiangsu 210003, P.R. China
[10]Guangdong Provincial Hospital of Chinese Medicine, The Second Affiliated Hospital of Guangzhou University of Chinese Medicine, Guangzhou, Guangdong 510405, P.R. China
[11]Hepatology Unit, Department of Infectious Diseases, Nanfang Hospital, Southern Medical University, Guangzhou, Guangdong 510925, P.R. China
[12]The First Affiliated Hospital of Xinjiang Medical University, Urumqi, Xinjiang 830054, P.R. China
[13]Department of Hepatology, Tianjin Second People's Hospital, Tianjin 301799, P.R. China
[14]Department of Hepatology, Beijing Youan Hospital, Capital Medical University, Beijing 100000, P.R. China
[15]The First Hospital of China Medical University, Shenyang, Liaoning 110001, P.R. China
[16]Center for Infectious Diseases, West China Hospital, Sichuan University, Chengdu Sichuan, P.R. China
[17]Center of Liver Diseases, Beijing Ditan Hospital, Capital Medical University, Beijing 100015, P.R. China
[18]Department of Gastroenterology, the First Affiliated Hospital, Sun Yat-sen University, Guangzhou, Guangdong 510062, P.R. China
[19]Karsh Division of Gastroenterology and Hepatology, Department of Medicine, Cedars-Sinai Medical Center, Los Angeles, CA 90048, USA
[20]Department of Endocrinology, Shanghai Fifth People's Hospital, Fudan University, Shanghai 200240, P.R. China
[21]These authors contributed equally
[22]Lead contact
*Correspondence: lijier@nju.edu.cn (J.L.), sophiazsf@fudan.edu.cn (S.Z.), 20131004@hznu.edu.cn (J.S.)
https://doi.org/10.1016/j.xcrm.2025.101939

SUMMARY

The efficacy and safety of a lower dose of vitamin E for metabolic dysfunction-associated steatohepatitis (MASH) treatment are unclear. This multi-center, randomized, double-blind, placebo-controlled study includes 124 non-diabetic participants with biopsy-proven MASH. Participants are randomly assigned to receive oral vitamin E 300 mg or the placebo in a 1:1 ratio. The primary outcome is improvement in hepatic histology. In the modified intention-to-treat population, 29.3% of participants in the vitamin E group achieve the primary outcome compared with 14.1% in the placebo group. Significant improvement in steatosis, lobular inflammation, and fibrosis stages is observed in the vitamin E group. 12 serious adverse events are reported in this trial but are not considered to be related to the treatment. Vitamin E 300 mg daily achieves sound improvements in liver histology in the Chinese population with MASH. This study is registered at ClinicalTrials.gov (NCT02962297).

© 2025 The Author(s). Published by Elsevier Inc.
This is an open access article under the CC BY license (http://creativecommons.org/licenses/by/4.0/).

INTRODUCTION

Metabolic dysfunction-associated steatotic liver disease (MASLD), previously termed non-alcoholic fatty liver disease (NAFLD), has become one of the most common chronic liver diseases worldwide, with prevalence approaching 30% both in the globe and in Asia.[1,2] Its more severe form, metabolic dysfunction-associated steatohepatitis (MASH), is characterized by hepatocellular damage and inflammation, with or without fibrosis, resulting in a heavier burden of liver-related morbidity and mortality. In 2024, the thyroid hormone receptor-β selective agonist resmetirom has been approved for the treatment of moderate-to-advanced fibrotic MASH.[3] However, the development of MASH is driven by various pathophysiological factors. There is still an unmet need for treatment in a large population with MASH and liver fibrosis.

Oxidative stress is the main contributor in the process of MASH. It occurs when the scavenging capacity of the antioxidant system exceeds the production of reactive oxygen species.[4–6] Vitamin E, a natural antioxidant, is one of the most promising treatments for MASH. Two landmark randomized clinical trials, PIVENS[7] and TONIC[8] studies, proved the efficacy of vitamin E both in adults and children. Since then, trials of vitamin E with different doses were conducted in adults with MASLD over the past decade, showing positive results in reduction of liver functions and liver histologic parameters.[9] However, the optimal dose of vitamin E is still inconclusive. An umbrella review of meta-analysis showed that prescription of less than 600 IU/day vitamin E can improve liver enzymes and liver steatosis, while significant liver fibrosis improvement can be achieved in vitamin E over 600 IU/day and when treatment duration is over 12 months[10] However, dose escalation of vitamin E raises safety concerns, including hemorrhagic stroke,[11] prostate cancer,[12,13] cardiovascular disease,[14] and even all-cause mortality.[15,16] Exploring an appropriate dose of vitamin E to balance the benefits and adverse effects is required.

In China, 300–450 IU/day (200–300 mg) of vitamin E is recommended for the Chinese population.[17] Previous pharmacokinetics analysis of vitamin E demonstrated that multiple doses of 300 mg daily showed comparable maximum concentration (C_{max}) and area under the curve with 600–800 mg daily administration.[18] Therefore, this multi-center, double-blind, randomized, placebo-controlled, investigator-initiated trial aimed to evaluate the efficacy and safety of vitamin E 300 mg versus the placebo for the treatment of non-diabetic participants with MASH (VENS).

RESULTS

Participants

A total of 169 participants were screened between January 24, 2017, and November 6, 2018, and 124 eligible participants were randomly assigned to the vitamin E 300 mg group and the placebo group in a 1:1 ratio, and they were considered as the full analysis set (FAS). Two participants were excluded from the modified intention-to-treat (mITT) population prior to unblinding agreed by the data monitoring committee as their baseline biopsy had been performed more than 6 months before enrollment. Finally, the mITT comprised 122 participants, and the safety analysis set included 124 based on inclusion and exclusion criteria (Figure 1).

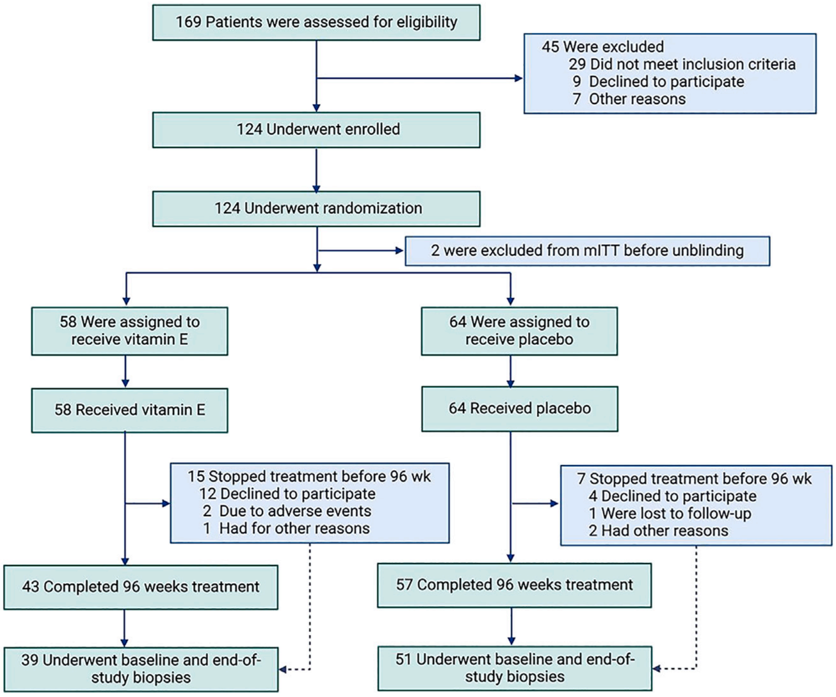

Figure 1. Flow chart

Table 1. Baseline characteristics of patients in the full analysis set (FAS)

	Vitamin E 300 mg group (*n* = 58)	Placebo group (*n* = 66)	*p* value
Demographic factors	–	–	–
Age, y	37.9 (11.7)	39.0 (11.6)	0.60
Gender	–	–	–
Male	42 (72.4%)	50 (75.8%)	–
Female	16 (27.6%)	16 (24.2%)	0.60
HP genotype	–	–	1.00
HP 1-1	0 (0.0%)	0 (0.0%)	–
HP 2-1	1 (1.7%)	2 (3.0%)	–
HP 2-2	57 (98.3%)	64 (97.0%)	–
Anthropometric parameters	–	–	–
BMI, kg/m^2	26.7 (4.1)	25.4 (2.8)	0.02
WHR	0.9 (0.1)	0.9 (0.0)	0.73
Serum biochemical levels	–	–	–
ALT, U/L	60.4 (36.8)	60.1 (37.8)	0.93
AST, U/L	33.6 (15.4)	36.0 (16.9)	0.51
γ-GT, U/L	44.7 (28.6)	46.0 (34.7)	0.60
AKP, U/L	85.8 (26.8)	84.5 (29.0)	0.60
TBIL, μmol/L	16.4 (9.7)	17.9 (8.5)	0.30
Lipids	–	–	–
TG, mmol/L	1.8 (0.9)	1.8 (1.0)	0.80
TC, mmol/L	4.6 (0.9)	4.6 (0.7)	0.54
HDL, mmol/L	1.2 (0.3)	1.1 (0.2)	0.82
LDL, mmol/L	2.9 (0.7)	2.8 (0.7)	0.92
Metabolic factors	–	–	–
FPG, mmol/L	5.2 (0.6)	5.3 (0.6)	0.85
FINS, pmol/L	92.3 (54.1)	94.9 (80.0)	0.61
HOMA-IR	3.6 (2.3)	3.7 (3.2)	0.61
2 h FPG, mmol/L	7.0 (1.9)	7.1 (2.1)	0.81
Proinflammatory cytokines	–	–	–
TNF-α, pg/mL	7.9 (0.6)	7.8 (0.6)	0.66
IL-6, pg/mL	5.1 (0.8)	5.2 (0.8)	0.34
Cytokeratin 18	–	–	–
Fragment M30, U/L	474.7 (47.8)	473.8 (41.9)	0.84
Fragment M65, U/L	739.5 (73.1)	727.6 (75.3)	0.55
FibroScan assessment	–	–	–
CAP, dB/m	278.8 (52.6)	278.4 (48.3)	0.44
LSM, kPa	7.7 (3.2)	6.5 (2.3)	0.06
Liver histology	–	–	–
Steatosis (%)	–	–	0.89
1	18 (31.0)	22 (33.3)	–
2	25 (43.1)	27 (40.9)	–
3	15 (25.9)	17 (25.8)	–
Lobular inflammation (%)	–	–	0.92
0	0 (0.0)	1 (1.5)	–
1	23 (39.7)	29 (44.0)	–
2	26 (44.8)	28 (42.4)	–
3	9 (15.5)	8 (12.1)	–

(*Continued on next page*)

Table 1. *Continued*

	Vitamin E 300 mg group (n = 58)	Placebo group (n = 66)	p value
Ballooning degeneration (%)	–	–	0.26
0	9 (15.5)	10 (15.2)	–
1	36 (62.1)	48 (72.7)	–
2	13 (22.4)	8 (12.1)	–
Total NAS (%)	–	–	0.59
2	4 (6.9)	6 (9.1)	–
3	8 (13.8)	7 (10.6)	–
4	11 (19.0)	17 (25.8)	–
5	18 (31.0)	19 (28.8)	–
6	10 (17.2)	15 (22.7)	–
7	5 (8.6)	1 (1.5)	–
8	2 (3.5)	1 (1.5)	–
Fibrosis (%)	–	–	0.41
0	28 (48.3)	25 (37.9)	–
1	21 (36.2)	28 (42.4)	–
2	6 (10.3)	10 (15.2)	–
3	3 (5.2)	2 (3.0)	–
4	0 (0.0)	1 (1.5)	–

Continuous variables are presented with mean (standard deviations), and categorical variable are presented with n (%). Abbreviations: Hp, haptoglobin; BMI, body mass index, calculated as weight in kilograms divided by height in meters squared; WHR, waist-to-hip ratio, calculated as waist circumference in meters divided by hip circumference in meters; ALT, alanine aminotransferase; AST, aspartate aminotransferase; γ-GT, glutamyl peptidyl transferase; AKP, alkaline phosphatase; TBIL, total bilirubin; TG, total triglycerides; TC, total cholesterol; LDL, low-density lipoprotein; HDL, high-density lipoprotein; FPG, fasting plasma glucose; FINS, fasting insulin; HOMA-IR, homeostasis model assessment for insulin resistance, calculated as insulin × glucose/22.5, where the unit of measure for insulin is μIU/mL and the unit of measure for glucose is mmol/L; TNF-α, tumor necrosis factor α; IL-6, interleukin-6; CAP, controlled attenuation parameter; LSM, liver stiffness measurement; NAS, non-alcoholic fatty liver disease activity score.

All of these participants received personalized lifestyle advice by dieticians during the trial (Figure S1). Study groups were well balanced for demographic and clinical characteristics. The mean (standard deviation [SD]) age was 37.9 (11.7) years in the vitamin E 300 mg group and 39.0 (11.6) years in the placebo group, with males accounting for 42 (72.4%) and 50 (75.8%), respectively. The corresponding mean (SD) body mass index (BMI) was 26.7 (4.1) kg/m^2 and 25.4 (2.8) kg/m^2 in the vitamin E 300 mg and the placebo groups, respectively (Table 1).

Hepatic histological parameters were well balanced between the two groups. 79.3% and 80.3% of participants in the vitamin E 300 mg and the placebo groups had an NAFLD activity score (NAS) score ≥4 at the final central reading (Table 1). The alanine aminotransferase (ALT) and aspartate aminotransferase (AST) levels in the vitamin E 300 mg group were 60.4 (36.8) U/L and 33.6 (15.4) U/L, respectively, and 60.1 (37.8) U/L and 36.0 (16.9) U/L in the placebo group, respectively. The liver stiffness measurement (LSM) was 7.7 (3.2) kPa in the vitamin E 300 mg group and 6.5 (2.3) kPa in the placebo group. As for the haptoglobin (HP) genotype, 98.3% of participants in the vitamin E 300 mg group and 97.0% of participants in the placebo group showed an HP 2-2 genotype. Only three participants were HP 2-1 genotype (one in the vitamin E and two in the placebo groups), and none were HP 1-1 genotype (Table 1).

Efficacy

The percentage of participants with liver histological improvement (the primary endpoint) after 96 weeks was significantly higher in the vitamin E 300 mg group than in the placebo group (29.3% vs. 14.1%, respectively; odds ratio [OR], 2.5; 95% confidence interval [CI]: 1.0–7.1; p = 0.04) (Figure 2A). Similar results were seen in sensitivity analyses when baseline biopsy specimens were at local reading and post 96-week specimens were at central reading (Table S1). Subgroup analyses for the primary outcome showed that males, participants aged below 40 years, and participants with NAS 5–8 in the vitamin E 300 mg group were associated with a significant histological improvement than those in the placebo group (Table S2). Typical pictures with improvements in liver histology were shown in Figure S2.

The difference between the vitamin E 300 mg treatment and the placebo groups in fibrosis improvement by at least one stage without worsening of steatohepatitis after 96 weeks (the exploratory secondary endpoint) did not reach statistical significance (25.9% vs. 15.6%, respectively; OR, 1.9; 95% CI: 0.7–5.2; p = 0.16) (Figure 2B). In addition, 12.1% of the participants in the vitamin E 300 mg group reached steatohepatitis resolution without worsening of fibrosis, whereas the percentage in the placebo group was 17.2% (OR, 0.7; 95% CI: 0.2–2.0; p = 0.43) (Figure 2C). The composite endpoint of fibrosis improvement by at least one stage or steatohepatitis resolution without fibrosis worsening also failed to achieve statistical significance between

CellPress
OPEN ACCESS

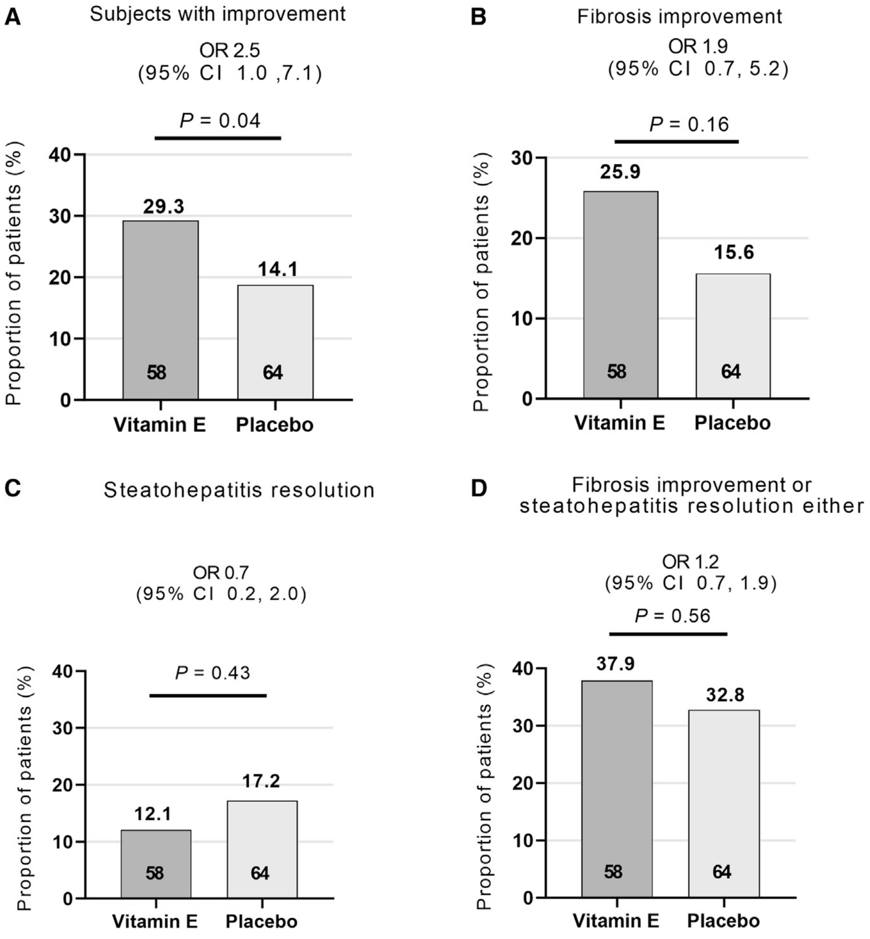

Figure 2. Primary endpoints and secondary endpoints (mITT)

(A) Subjects with liver histological improvement.
(B) Fibrosis improvement by at least one stage without worsening of steatohepatitis.
(C) Steatohepatitis resolution without worsening of fibrosis.
(D) Fibrosis improvement by ≥1 stage or steatohepatitis resolution without fibrosis worsening either.
Data are presented as percentage (%). See also Tables S1 and S2.

the vitamin E 300 mg and the placebo groups (37.9% vs. 32.8%, respectively; OR, 1.2; 95% CI: 0.7–1.9; p = 0.56) (Figure 2D).

For each component of NAS, participants who received vitamin E 300 mg showed a significant reduction in steatosis (win ratio [WR]: 2.5, 95% CI: 1.3–5.0, p = 0.01), lobular inflammation (WR: 2.0, 95% CI: 1.0–4.0, p = 0.04), fibrosis score (WR: 2.1, 95% CI: 1.0–4.0, p = 0.04), and total NAS score (WR: 1.9, 95% CI: 1.1–3.4, p = 0.03) compared with the placebo group, while hepatocyte ballooning did not achieve significant improvement between the two groups (Figure 3). Proportion changes in histologic features were listed in Table S3.

There was a rapid decrease in ALT and AST in both groups at 24 weeks, but only in those who received vitamin E 300 mg had a sustained decrease at 96-week post-treatment and 24-week follow-up (Figure 4 and Table S4). As for the controlled attenuation parameter (CAP) and LSM, favorable and stable mean (SD) changes from baseline to 96 weeks were observed in the vitamin E 300 mg group compared to the placebo group (CAP: −6.4 [56.8] vs. 8.3 [54.2], p = 0.06; LSM: −1.2 [3.2] vs. 0.3 [2.4], p = 0.04) (Table 2). In addition, proinflammatory cytokines interleukin-6 (IL-6) decreased significantly in the vitamin E 300 mg group compared with the placebo group (−0.1 [0.7] vs. 0.3 [0.7], p = 0.04). The level of tumor necrosis factor-α (TNF-α) showed the downward trend in the vitamin E 300 mg group (Table 2). Changes to lipids and glucose profiles did not differ significantly between the two groups (Table 2).

Safety

A total of 124 participants were included in the safety population. There were 11 adverse events, with seven (12.07%) in the vitamin E group and four (6.06%) in the placebo group (Table 3). Within the 96-week intervention time frame, the incidence rate was 7.17 cases and 4.21 cases per 100 person-years in the vitamin E and placebo groups, respectively. However, they were not considered treatment-related adverse events. Combination therapies were listed in Table S5. There were no instances of cardiovascular events, heart failure, or new-onset diabetes during the intervention and after 24 weeks of follow-up. In males, serum brain natriuretic peptide (BNP) and prostate-specific antigen (PSA) remained within the normal range, and prostate ultrasound examinations for male participants revealed no abnormality after 96 weeks of treatment (Figure S3).

DISCUSSION

This study assessed the liver histological efficacy of 300 mg vitamin E in participants with biopsy-proven MASH over a 96-week intervention period, followed by a 24-week follow-up. Our study showed that a low dosage of vitamin E successfully achieved the primary endpoint, significantly improving the overall liver histology. Remarkable reductions were also observed in steatosis, lobular inflammation, total NAS, and fibrosis. In addition, 300 mg vitamin E with 96-week treatment achieved significant improvement in liver enzymes and LSM.

Previous studies have proved that individuals with MASH could benefit from high dose of vitamin E. Harrison et al.[19] conducted a prospective clinical trial in individuals with MASH, demonstrating that the combination of 1,000 IU vitamin E and 1,000 mg vitamin C per day for 6 months significantly reduced biopsy-confirmed liver fibrosis score but without inflammation

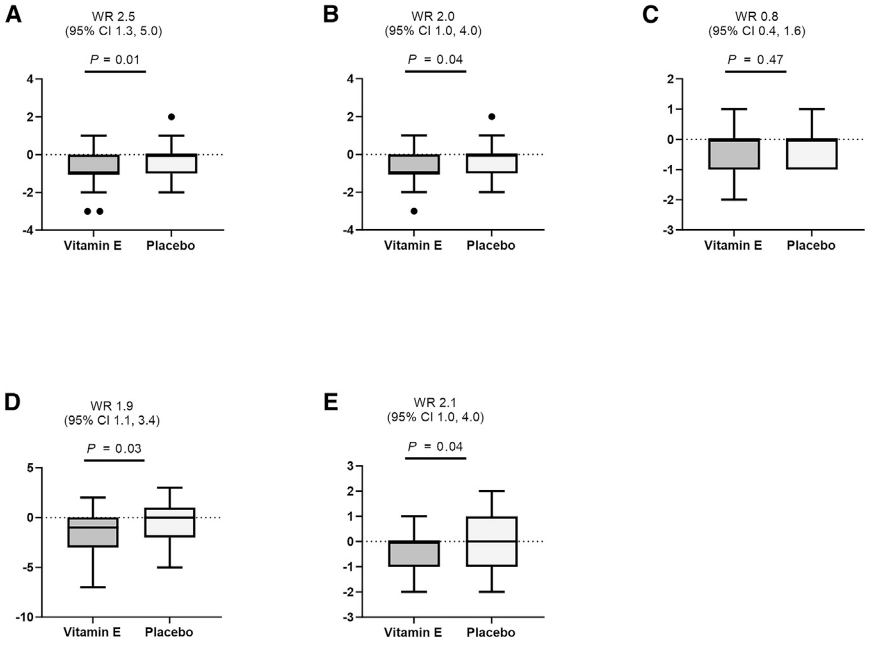

Figure 3. Changes from baseline in histologic features (mITT)

(A) Steatosis, (B) lobular inflammation, (C) hepatocyte ballooning, (D) total NAFLD activity score, and (E) fibrosis.

Data are presented as the inter-quartile range (IQR), and the upper and lower error bars represent 75th percentile plus 1.5 times IQR and the 25th percentile minus 1.5 IQR, respectively. NAFLD, non-alcoholic fatty liver disease. See also Table S3.

improvement. In 2010, the PIVENS study highlighted the efficacy of 800 IU vitamin E in the treatment of MASH.[7] Our study demonstrated that a relatively lower dosage of vitamin E was capable of improving liver histological parameters.

In this study, the definition of histological improvement was consistent with the PIVENS study.[7] A few participants in this study showed a baseline ballooning score of 0 and total NAS of 2–3 re-assessed by the central panel of pathologists. Therefore, an NAS decrease of 2 was considered a histological improvement. The response rate in our study was lower than that in the intention-to-treat (ITT) population reported in the PIVENS study, which may be partly due to relatively high missing data in this study. Nonetheless, the overall liver histological improvement rate in this study was comparable to the results of the per-protocol set in the PIVENS study.

The exploratory secondary endpoints, including an improvement in liver fibrosis by ≥1 stage without worsening of steatohepatitis, steatohepatitis resolution without liver fibrosis worsening, and improvement in fibrosis by ≥1 stage or steatohepatitis resolution without fibrosis worsening, did not achieve significant differences between the two groups. However, the vitamin E 300 mg group improved fibrosis in the mITT population, significantly higher than those in the placebo group. Moreover, significant change was observed in LSM evaluated by FibroScan at the end of the treatment in the vitamin E 300 mg group in comparison with the placebo group. Liver fibrosis, a crucial point for liver-related adverse outcomes, is important in the whole process of MASLD development. Although only a few pilot trials showed that vitamin E with a high dosage could change biopsy-confirmed liver fibrosis,[19,20] a retrospective study inclusive of participants with MASH and advanced fibrosis showed that treatment with vitamin E was associated with lower hepatic decompensation and higher transplant-free survival.[21] It was speculated that vitamin E has a hepatoprotective effect by reducing oxidative damage to liver cells, inhibiting *de novo* lipogenesis via decreasing sterol regulatory-element binding protein-1 processing, and lipogenic gene expression.[22] It was also found that vitamin E was associated with cholesterol homeostasis, inflammatory pathways, and cellular trafficking in a non-antioxidant manner.[23] However, it must be admitted that currently there is still a lack of robust data to support the benefits of vitamin E in the improvement of liver fibrosis under the criteria of the Food and Drug Administration. At the end of the 96-week intervention, significant changes

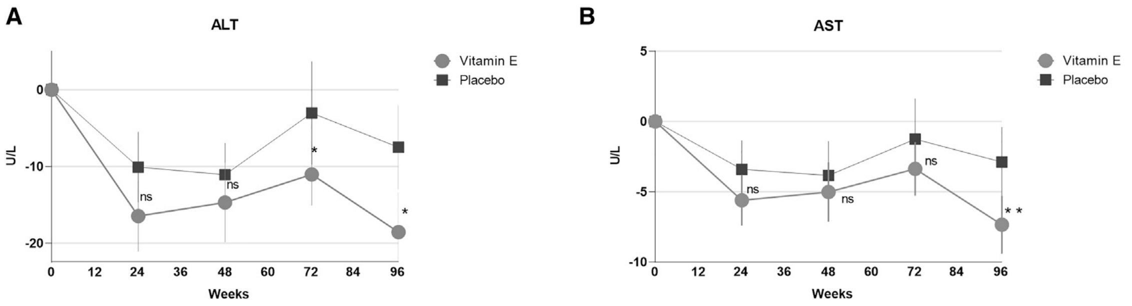

Figure 4. Change between baseline and week 96 in ALT and AST (FAS)

(A) ALT and (B) AST.

Data are presented as mean ± standard error. ALT, alanine aminotransferase; AST, aspartate aminotransferase.

CellPress
OPEN ACCESS

Table 2. Changes between baseline and week 96 in selective secondary endpoints (FAS)

	Vitamin E 300 mg group (*n* = 58)	Placebo group (*n* = 66)	Point estimate (95% CI)	*p* Value (ANCOVA)
Changes in anthropometric parameters	–	–	–	–
BMI, kg/m^2	0.8 (3.2)	0.0 (2.0)	1.1 (0.2, 2.0)	0.02
WHR[a]	0.0 (0.0)	0.0 (0.0)	0.0 (0.0, 0.0)	0.68
Liver enzyme	–	–	–	–
ALT, U/L	−22.2 (36.6)	−6.0 (44.3)	−16.0 (−27.1, −4.9)	0.01
AST, U/L	−8.8 (16.4)	−2.8 (19.6)	−7.9 (−12.4, −3.4)	<0.01
γ-GT, U/L	−3.3 (21.7)	0.4 (27.0)	−4.3 (−11.7, 3.0)	0.25
AKP, U/L	−2.2 (24.8)	−2.1 (24.0)	0.5 (−7.2, 8.1)	0.90
TBIL, μmol/L	−2.3 (8.0)	−1.9 (7.2)	−1.3 (−3.2, 0.6)	0.17
Lipids	–	–	–	–
TG, mmol/L	0.3 (1.2)	0.4 (1.4)	−0.2 (−0.7, 0.3)	0.48
TC, mmol/L	0.4 (1.1)	0.3 (1.0)	0.1 (−0.3, 0.4)	0.75
HDL, mmol/L	0.0 (0.3)	0.0 (0.2)	0.0 (−0.1, 0.1)	0.71
LDL, mmol/L	0.2 (0.8)	0.2 (0.8)	0.0 (−0.3, 0.2)	0.86
Metabolic factors	–	–	–	–
FPG[a], mmol/L	−0.1 (0.8)	0.2 (0.8)	−0.2 (−0.5, 0.0)	0.90
FINS, pmol/L	−14.8 (75.3)	−12.8 (75.3)	−2.0 (−28.9, 24.8)	0.86
HOMA-IR	−0.5 (3.4)	−0.4 (3.4)	-0.1 (−1.30, 1.11)	0.84
2 h blood glucose, mmol/L	0.5 (2.8)	0.6 (3.3)	−0.3 (−1.2, 0.7)	0.58
Changes in proinflammatory cytokines[b]	–	–	–	–
TNF-α, pg/mL	−0.8 (1.2)	−0.2 (0.4)	−0.4 (−1.4, 0.5)	0.33
IL-6, pg/mL	−0.1 (0.7)	0.3 (0.7)	−0.7 (−1.4, 0.0)	0.04
Cytokeratin 18[b]	–	–	–	–
Fragment M30, U/L	−81.0 (99.8)	−20.3 (88.3)	−60.6 (−93.6, −27.6)	<0.001
Fragment M65, U/L	−90.4 (120.4)	−22.5 (82.0)	−63.8 (−94.5, −33.0)	<0.001
FibroScan assessment	–	–	–	–
CAP, dB/m	−6.4 (56.8)	8.3 (54.2)	−14.4 (−29.1, 0.4)	0.06
LSM, kPa	−1.2 (3.2)	0.3 (2.4)	−0.9 (−1.8, 0.0)	0.04

Data are presented as mean (SD). An analysis of covariance (ANCOVA) model was used, with the absolute change from baseline as the dependent variable, treatment group as a fixed factor, and baseline value as a covariate.
BMI, body mass index, calculated as weight in kilograms divided by height in meters squared; WHR, waist-to-hip ratio, calculated as waist circumference in meters divided by hip circumference in meters; ALT, alanine aminotransferase; AST, aspartate aminotransferase; γ-GT, Glutamyl peptidyl transferase; AKP, alkaline phosphatase; TG, total triglycerides; TC, total cholesterol; LDL, low-density lipoprotein; HDL, high-density lipoprotein; FPG, fasting plasma glucose; FINS, fasting insulin; HOMA-IR, homeostasis model assessment for insulin resistance, calculated as insulin × glucose/22.5, where the unit of measure for insulin is μIU/mL and the unit of measure for glucose is mmol/L; TNF-α, tumor necrosis factor α; IL-6, interleukin-6; CAP, controlled attenuation parameter; LSM, liver stiffness measurement.
See also Table S4.
[a]Both baseline BMI and corresponding baseline value were included as covariates in the ANCOVA model considering the *p* value for the main effect of baseline BMI was less than 0.05.
[b]*n* = 39 in the vitamin E group and *n* = 52 in the placebo group.

in ALT and AST were observed in the vitamin E 300 mg group compared with the placebo group. In addition, IL-6, a representative proinflammatory cytokine, was also significantly lower in the vitamin E 300 mg group compared with the placebo group. However, no significant improvements were observed in lipid and glucose profiles in this study.

HP is a secretory protein synthesized by hepatocytes and binds to hemoglobin to prevent oxidative stress mediated by heme iron. The *HP* gene has two alleles, HP1 and HP2, giving rise to three genotypes (HP 1-1, HP 2-1, and HP 2-2). HP 2-2 protein has been reported to show lower antioxidant activity than other protein forms.[24,25] Unexpectedly, among participants enrolled in this trial, only three participants had genotype HP 2-1 and none had HP 1-1. The predominance of HP 2-2 in participants with MASH is one of the potential reasons for the efficacy of low-dose vitamin E.

For safety profiles, treatment with 300 mg vitamin E was well tolerated. No participants died during the study, and no serious adverse events were determined to be causally related to the study treatment. All adverse events resolved without sequelae following appropriate treatment. Prostate cancer, cardiovascular disease, and stroke with long-term and high-dose

Table 3. Clinical events, outcomes, and severe adverse events (safety set)

	Vitamin E 300 mg group (*n* = 58)	Placebo group (*n* = 66)	*p* value
Total adverse events	7 (12.07%)	4 (6.06%)	0.24
Kidney and urinary tract diseases	2 (3.45%)	1 (1.52%)	0.50
Chronic glomerulonephritis	1	0	–
Hydronephrosis	0	1	–
Ureteral calculi	1	0	–
All kinds of injuries, poisoning, and operational complications	1 (1.72%)	1 (1.52%)	0.94
Bone fracture	1	0	–
Tendon injury	0	0	–
Lumbar spine fracture	0	1	–
Benign, malignant, and tumors of unknown nature (including cystic and polypoid)	1 (1.72%)	1 (1.52%)	0.94
Bladder tumor	0	1	–
Adenocarcinoma of the lung	1	0	–
Infectious and invasive diseases	0 (0.00%)	1 (1.52%)	0.34
Infectious pneumonia	0	1	–
Various musculoskeletal and connective tissue disorders	1 (1.72%)	0 (0.00%)	0.29
Periarthritis	1	0	–
Various surgical and medical procedures	1 (1.72%)	0 (0.00%)	0.29
Induced abortion	1	0	–
Gastrointestinal system diseases	1 (1.72%)	0 (0.00%)	0.29
Colorectal polyps	1	0	–

Data are presented as *n* (%).

use of vitamin E have been a concern in previous studies.[13,16] In this trial, neither prostate cancer nor cardiovascular events or stroke developed during the 96-week intervention phase or 24-week follow-up phase. In addition, there were no abnormal results in BNP, PSA (for males), or the prostate (for males) evaluated by ultrasound after treatment.

Limitations of the study

This trial has several limitations. Firstly, shadowed by the pandemic of COVID-19, the dropout rate exceeded expectations and negatively impacted the efficacy evaluation. Since participants who were lost to follow-up were considered to have no response to treatment, the efficacy of vitamin E in the mITT population was lower than expectations. Secondly, all the participating centers were located in China, and the results in our study should be interpreted with caution when extrapolating to other countries and regions. Thirdly, the biopsy results were initially assessed at each participating center during recruitment, with the final baseline biopsy results determined by central reading. This process introduced variability in NAS and fibrosis evaluations, potentially biasing the final outcomes. Finally, the sample size was relatively small, potentially because liver biopsy is not accepted by most participants due to its invasiveness.

Conclusions

In summary, the study confirmed that 300 mg vitamin E with a 96-week treatment had positive effects on overall liver histology and key NAS components in participants with biopsy-proven MASH. This dosage of vitamin E also showed improvement in liver enzymes and proinflammatory cytokines without obvious safety concern. Vitamin E with 300 mg per day may offer a safer long-term treatment option for participants with MASH.

RESOURCE AVAILABILITY

Lead contact

Further information and requests for resources and reagents should be directed to and will be fulfilled by the lead contact, Junping Shi (20131004@hznu.edu.cn).

Materials availability

This study did not generate new unique reagents.

Data and code availability

- De-identified individual participant-level data required to reanalyze the data are available from the lead contact upon request. Any additional details about individual participants that could compromise patient confidentiality will not be disclosed.
- This paper does not report original code.
- Any additional information required to reanalyze the data reported in this paper is available from the lead contact upon request.

ACKNOWLEDGMENTS

We thank the independent pathologists who performed central biopsy evaluations (Shuyuan Xiao, Jingmin Zhao, and Wenjun Yang) and all the

participants, investigators, and trial staff involved in the conduct of the trial. This work was supported by grants from Noncommunicable Chronic Diseases-National Science and Technology Major Project (no. 2023ZD0508704 and no. 2023ZD0508802), Interdisciplinary Research Project of Hangzhou Normal University (2024JCXK06), the National Natural Science Foundation of China (no. 82170609 and 81970545), NSFC-RGC Forum for Young Scholars (no. 82411560273), the Natural Science Foundation of Jiangsu Province (no. BK20231118), and Zhejiang Medicine Co., Ltd.

AUTHOR CONTRIBUTIONS

J.L., S.Z., and J.S. were responsible for the study design in collaboration with the sponsor (S.X.). M.Z., H.S., J.W., S.X., Y.Y., X.C., J.C., F.H., X.F., Y.M., J.Z., B.W., L.B., W.X., B.Z., and J.S. (Chair) comprised the steering committee and were responsible for the conduct of the study. Y.S., W.N., F.R., S.X., R&G PharmaStudies (China) Co., Ltd, and Beijing SSYP Data Technology Development Co., Ltd participated in data collection, data analysis, and interpretation. W.N., S.Z., J.L., and Y.H.Y. were responsible for manuscript writing.

DECLARATION OF INTERESTS

The authors declare no competing interests.

STAR★METHODS

Detailed methods are provided in the online version of this paper and include the following:

- KEY RESOURCES TABLE
- EXPERIMENTAL MODEL AND STUDY PARTICIPANT DETAILS
- METHOD DETAILS
  - Randomization and intervention
  - Liver biopsies
  - Outcomes
  - Laboratory testing
  - Recommendations for diet and exercise
- QUANTIFICATION AND STATISTICAL ANALYSES
- ADDITIONAL RESOURCES

SUPPLEMENTAL INFORMATION

Supplemental information can be found online at https://doi.org/10.1016/j.xcrm.2025.101939.

Received: September 14, 2024
Revised: November 15, 2024
Accepted: January 13, 2025
Published: February 18, 2025

REFERENCES

1. Le, M.H., Yeo, Y.H., Li, X., Li, J., Zou, B., Wu, Y., Ye, Q., Huang, D.Q., Zhao, C., Zhang, J., et al. (2022). 2019 Global NAFLD Prevalence: A Systematic Review and Meta-analysis. Clin. Gastroenterol. Hepatol. *20*, 2809–2817.e28. https://doi.org/10.1016/j.cgh.2021.12.002.
2. Li, J., Zou, B., Yeo, Y.H., Feng, Y., Xie, X., Lee, D.H., Fujii, H., Wu, Y., Kam, L.Y., Ji, F., et al. (2019). Prevalence, incidence, and outcome of non-alcoholic fatty liver disease in Asia, 1999-2019: a systematic review and meta-analysis. Lancet. Gastroenterol. Hepatol. *4*, 389–398. https://doi.org/10.1016/S2468-1253(19)30039-1.
3. Harrison, S.A., Bedossa, P., Guy, C.D., Schattenberg, J.M., Loomba, R., Taub, R., Labriola, D., Moussa, S.E., Neff, G.W., Rinella, M.E., et al. (2024). A Phase 3, Randomized, Controlled Trial of Resmetirom in NASH with Liver Fibrosis. N. Engl. J. Med. *390*, 497–509. https://doi.org/10.1056/NEJMoa2309000.
4. Chen, Z., Tian, R., She, Z., Cai, J., and Li, H. (2020). Role of oxidative stress in the pathogenesis of nonalcoholic fatty liver disease. Free Radic. Biol. Med. *152*, 116–141. https://doi.org/10.1016/j.freeradbiomed.2020.02.025.
5. Clare, K., Dillon, J.F., and Brennan, P.N. (2022). Reactive Oxygen Species and Oxidative Stress in the Pathogenesis of MAFLD. J. Clin. Transl. Hepatol. *10*, 939–946. https://doi.org/10.14218/JCTH.2022.00067.
6. Friedman, S.L., Neuschwander-Tetri, B.A., Rinella, M., and Sanyal, A.J. (2018). Mechanisms of NAFLD development and therapeutic strategies. Nat. Med. *24*, 908–922. https://doi.org/10.1038/s41591-018-0104-9.
7. Sanyal, A.J., Chalasani, N., Kowdley, K.V., McCullough, A., Diehl, A.M., Bass, N.M., Neuschwander-Tetri, B.A., Lavine, J.E., Tonascia, J., Unalp, A., et al. (2010). Pioglitazone, vitamin E, or placebo for nonalcoholic steatohepatitis. N. Engl. J. Med. *362*, 1675–1685. https://doi.org/10.1056/NEJMoa0907929.
8. Lavine, J.E., Schwimmer, J.B., Van Natta, M.L., Molleston, J.P., Murray, K.F., Rosenthal, P., Abrams, S.H., Scheimann, A.O., Sanyal, A.J., Chalasani, N., et al. (2011). Effect of vitamin E or metformin for treatment of nonalcoholic fatty liver disease in children and adolescents: the TONIC randomized controlled trial. JAMA *305*, 1659–1668. https://doi.org/10.1001/jama.2011.520.
9. Vadarlis, A., Antza, C., Bakaloudi, D.R., Doundoulakis, I., Kalopitas, G., Samara, M., Dardavessis, T., Maris, T., and Chourdakis, M. (2021). Systematic review with meta-analysis: The effect of vitamin E supplementation in adult patients with non-alcoholic fatty liver disease. J. Gastroenterol. Hepatol. *36*, 311–319. https://doi.org/10.1111/jgh.15221.
10. Wang, M.Y., Prabahar, K., Găman, M.A., and Zhang, J.L. (2023). Vitamin E supplementation in the treatment on nonalcoholic fatty liver disease (NAFLD): Evidence from an umbrella review of meta-analysis on randomized controlled trials. J Dig Dis *6–7*, 380–389. https://doi.org/10.1111/1751-2980.13210.
11. Schürks, M., Glynn, R.J., Rist, P.M., Tzourio, C., and Kurth, T. (2010). Effects of vitamin E on stroke subtypes: meta-analysis of randomised controlled trials. BMJ *341*, c5702. https://doi.org/10.1136/bmj.c5702.
12. Gaziano, J.M., Sesso, H.D., Christen, W.G., Bubes, V., Smith, J.P., MacFadyen, J., Schvartz, M., Manson, J.E., Glynn, R.J., and Buring, J.E. (2012). Multivitamins in the prevention of cancer in men: the Physicians' Health Study II randomized controlled trial. JAMA *308*, 1871–1880. https://doi.org/10.1001/jama.2012.14641.
13. Klein, E.A., Thompson, I.M., Jr., Tangen, C.M., Crowley, J.J., Lucia, M.S., Goodman, P.J., Minasian, L.M., Ford, L.G., Parnes, H.L., Gaziano, J.M., et al. (2011). Vitamin E and the risk of prostate cancer: the Selenium and Vitamin E Cancer Prevention Trial (SELECT). JAMA *306*, 1549–1556. https://doi.org/10.1001/jama.2011.1437.
14. US Preventive Services Task Force, Mangione, C.M., Barry, M.J., Nicholson, W.K., Cabana, M., Chelmow, D., Coker, T.R., Davis, E.M., Donahue, K.E., Doubeni, C.A., et al. (2022). Vitamin, Mineral, and Multivitamin Supplementation to Prevent Cardiovascular Disease and Cancer: US Preventive Services Task Force Recommendation Statement. JAMA *327*, 2326–2333. https://doi.org/10.1001/jama.2022.8970.
15. Abner, E.L., Schmitt, F.A., Mendiondo, M.S., Marcum, J.L., and Kryscio, R.J. (2011). Vitamin E and all-cause mortality: a meta-analysis. Curr. Aging Sci. *4*, 158–170. https://doi.org/10.2174/1874609811104020158.
16. Miller, E.R., 3rd, Pastor-Barriuso, R., Dalal, D., Riemersma, R.A., Appel, L.J., and Guallar, E. (2005). Meta-analysis: high-dosage vitamin E supplementation may increase all-cause mortality. Ann. Intern. Med. *142*, 37–46. https://doi.org/10.7326/0003-4819-142-1-200501040-00110.
17. Zhou, Q., Su, J., and JI, M.Y. (2020). Progress in the treatment of nonalcoholic fatty liver disease. China Med Herald 6, 26–29.
18. Mahipal, A., Klapman, J., Vignesh, S., Yang, C.S., Neuger, A., Chen, D.T., and Malafa, M.P. (2016). Pharmacokinetics and safety of vitamin E δ-tocotrienol after single and multiple doses in healthy subjects with

measurement of vitamin E metabolites. Cancer Chemother. Pharmacol. *78*, 157–165. https://doi.org/10.1007/s00280-016-3048-0.

19. Harrison, S.A., Torgerson, S., Hayashi, P., Ward, J., and Schenker, S. (2003). Vitamin E and vitamin C treatment improves fibrosis in patients with nonalcoholic steatohepatitis. Am. J. Gastroenterol. *98*, 2485–2490. https://doi.org/10.1111/j.1572-0241.2003.08699.x.
20. Sumida, Y., Naito, Y., Tanaka, S., Sakai, K., Inada, Y., Taketani, H., Kanemasa, K., Yasui, K., Itoh, Y., Okanoue, T., and Yoshikawa, T. (2013). Long-term (>= 2 yr) efficacy of vitamin E for non-alcoholic steatohepatitis. Hepato-Gastroenterology *60*, 1445–1450. https://doi.org/10.5754/hge11421.
21. Vilar-Gomez, E., Vuppalanchi, R., Gawrieh, S., Ghabril, M., Saxena, R., Cummings, O.W., and Chalasani, N. (2020). Vitamin E Improves Transplant-Free Survival and Hepatic Decompensation Among Patients With Nonalcoholic Steatohepatitis and Advanced Fibrosis. Hepatology *71*, 495–509. https://doi.org/10.1002/hep.30368.
22. Podszun, M.C., Alawad, A.S., Lingala, S., Morris, N., Huang, W.C.A., Yang, S., Schoenfeld, M., Rolt, A., Ouwerkerk, R., Valdez, K., et al. (2020). Vitamin E treatment in NAFLD patients demonstrates that oxidative stress drives steatosis through upregulation of de-novo lipogenesis. Redox Biol. *37*, 101710. https://doi.org/10.1016/j.redox.2020.101710.
23. Hadi, H., Vettor, R., and Rossato, M. (2018). Vitamin E as a Treatment for Nonalcoholic Fatty Liver Disease: Reality or Myth? Antioxidants *7*, 12. https://doi.org/10.3390/antiox7010012.
24. Bamm, V.V., Tsemakhovich, V.A., Shaklai, M., and Shaklai, N. (2004). Haptoglobin phenotypes differ in their ability to inhibit heme transfer from hemoglobin to LDL. Biochemistry *43*, 3899–3906. https://doi.org/10.1021/bi0362626.
25. Melamed-Frank, M., Lache, O., Enav, B.I., Szafranek, T., Levy, N.S., Ricklis, R.M., and Levy, A.P. (2001). Structure-function analysis of the antioxidant properties of haptoglobin. Blood *98*, 3693–3698. https://doi.org/10.1182/blood.v98.13.3693.
26. Zang, S., Chen, J., Song, Y., Bai, L., Chen, J., Chi, X., He, F., Sheng, H., Wang, J., Xie, S., et al. (2018). Haptoglobin Genotype and Vitamin E Versus Placebo for the Treatment of Nondiabetic Patients with Nonalcoholic Steatohepatitis in China: A multi-center, Randomized, Placebo-Controlled Trial Design. Adv. Ther. *35*, 218–231. https://doi.org/10.1007/s12325-018-0670-8.
27. Banini, B.A., Cazanave, S.C., Yates, K.P., Asgharpour, A., Vincent, R., Mirshahi, F., Le, P., Contos, M.J., Tonascia, J., Chalasani, N.P., et al. (2019). Haptoglobin 2 Allele is Associated With Histologic Response to Vitamin E in Subjects With Nonalcoholic Steatohepatitis. J. Clin. Gastroenterol. *53*, 750–758. https://doi.org/10.1097/MCG.0000000000001142.
28. Zhou, J., Liu, J., Sheng, H., You, N., Chen, J., Mi, X., Yang, W., Zang, S., and Shi, J.; Chinese NAFLD Clinical Research Network CNAFLD CRN (2019). Haptoglobin 2-2 Genotype is Associated with More Advanced Disease in Subjects with Non-Alcoholic Steatohepatitis: A Retrospective Study. Adv. Ther. *36*, 880–895. https://doi.org/10.1007/s12325-019-00902-z.
29. Zi, W., Song, J., Kong, W., Huang, J., Guo, C., He, W., Yu, Y., Zhang, B., Geng, W., Tan, X., et al. (2023). Tirofiban for Stroke without Large or Medium-Sized Vessel Occlusion. N. Engl. J. Med. *388*, 2025–2036. https://doi.org/10.1056/NEJMoa2214299.
30. Wang, D., and Pocock, S. (2016). A win ratio approach to comparing continuous non-normal outcomes in clinical trials. Pharm. Stat. *15*, 238–245. https://doi.org/10.1002/pst.1743.

CellPress
OPEN ACCESS

STAR★METHODS

KEY RESOURCES TABLE

REAGENT or RESOURCE	SOURCE	IDENTIFIER
Biological samples		
Blood and liver biopsy samples of the participants	this study	N/A
Critical commercial assays		
QIAamp DNA Blood Kit	Qiagen	#51104
Software and algorithms		
SAS version 9.4	SAS Institute	https://www.sas.com/
R version 4.2.3	R-Project	https://www.r-project.org/
GraphPad Prism version 10.1.2	GraphPad Prism	https://www.graphpad.com/scientificsoftware/prism/
Biorender	Biorender	biorender.com

EXPERIMENTAL MODEL AND STUDY PARTICIPANT DETAILS

The VENS trial was a multi-center, double-blind, randomized, placebo-controlled, investigator-initiated trial performed at 14 clinical centers in Mainland of China (NCT02962297), including Hangzhou Normal University Affiliated Hospital; Beijing Youan Hospital, Capital Medical University; Tianjin Second People's Hospital; Nanfang Hospital, Southern Medical University; Guangdong Provincial Hospital of Chinese Medicine; The First Hospital of China Medical University; The Second Hospital of Nanjing, Affiliated to Nanjing University of Chinese Medicine; West China Hospital, Sichuan University; The Affiliated Traditional Chinese Medicine Hospital of Southwest Medical University; The First Affiliated Hospital of Xinjiang Medical University; General Hospital of Ningxia Medical University; The First Affiliated Hospital of Wenzhou Medical University; Beijing Ditan Hospital, Capital Medical University; The First Affiliated Hospital, Sun Yat-sen University. The study was approved by the ethics committee at each investigational center. All participants provided written informed consent.

The inclusion criteria were followed as: (1) Age: 18–75, no limitation for ethnic and gender; (2) BMI <35 kg/m^2; (3) Patients with NASH based on liver biopsy obtained within 6 months before randomization. The histological evidence of NASH was defined as NAS $\geq$4 (according to the criteria of the Nonalcoholic Steatohepatitis Clinical Research Network [NASH CRN]) at each center for local reading with a minimum score of 1 for steatosis, lobular inflammation, and hepatocyte ballooning, respectively[26]; (4) Fibrosis stage 0–3 according to NASH CRN[26]; (5) Without a history of significant alcohol consumption for a period of more than 3 months within 5 years (<10 g/day for females and <20 g/day for males); (6) The laboratory test results should meet the requirements: ① ALT <5 times of normal upper limit, ② Creatinine < normal upper limit; ③ Albumin >3.5 g/L; ④ International normalized ratio = 0.8–1.3; ⑤ Fasting plasma glucose <126 mg/dL (7 mmol/L) and/or 2h postprandial plasma glucose <200 mg/dL (11.1 mmol/L) and/or glycated hemoglobin <6.5%; (7) If a participant with hypertension, he/she was required a stable antihypertensive drug(s) to keep a stable blood pressure (blood pressure <140/90 mmHg) 3 months prior to randomization; (8) If a participant using a statin or fibrate, he/she was required on a stable dose to keep lipid stable (triglyceride <1.7 mmol/L, total cholesterol <5.72 mmol/L, low-density lipoprotein< 3.64 mmol/L) 3 months prior to randomization; (9) Women of childbearing potential: negative pregnancy test during screening or at randomization or willingness to use an effective form of birth control during the trial (at least include one barrier contraceptive method) and not breast feeding; (10) Men must agree to use an effective form of birth control during the trial (at least include one barrier contraceptive method); (11) All participants were needed to sign the informed consent form.

The exclusion criteria were followed as: (1) Evidence of other form of acute or chronic liver disease (virus hepatitis, hereditary hemochromatosis, hepatolenticular degeneration, alcoholic liver disease, drug-induced hepatopathy); (2) History of diabetic mellitus or use of antidiabetic drugs; (3) Known heart failure of New York Heart Association class 2, 3, or 4; (4) Wear of cardiac pacemaker; (5) Hypothyroidism (thyroid-stimulating hormone >2 times of upper normal limit); (6) History of disease affecting drug absorption, distribution, metabolism (inflammatory bowel disease, gastrointestinal surgery, chronic pancreatitis, gluten allergy, vagotomy); (7) Use of drugs indicating potential anti-steatohepatitis and/or fibrosis effects within 3 months before randomization (metformin, thiazolidinediones, dipeptidyl peptidase-4 inhibitor, glucagon-like peptide-1, sodium glucose contransporter 2 inhibitor, S-adenosylmethionine-e, polyene phosphatidyl choline, glycyrrhizin, bicyclol, reduced glutathione, betaine, fish oil, silymarin, oberbic acid/ursodeoxycholic acid, phosphodiesterase-inhibitor, gemfibrozil, vitamin E, long term antibiotic (>1 week); (8) Positivity of antibody to human immunodeficiency virus; (9) Inability to safely obtain liver biopsy; (10) Known intolerance to vitamin E; (11) Inability to fill out diary card, to manage diet and exercise, poor compliance; (12) Dependence or abuse of alcohol and/or drugs; (13) Any other condition which in the opinion of investigator would impede compliance or hinder completion of the study.

Finally, a total of 124 participants were recruited in the FAS. The mean (SD) age in the vitamin E 300 mg and the placebo groups was 37.9 (11.7) years and 39.0 (11.6) years, respectively. The proportion of males was 72.4% in the vitamin E 300 mg group and 75.8% in the placebo group.

METHOD DETAILS

Randomization and intervention

Participants were randomized in a 1:1 ratio to receive either vitamin E (natural RRR-α-tocopherol, produced by Zhejiang Medicine Co., Ltd, lot No. of production 160920, 16091201, 19010101) 300 mg (100 mg three times per day) or the placebo with same dose and frequency. The vitamin E capsule contained RRR-α-tocopherol, refined corn oil, gelatin, glycerin, and purified water, with a caloric content of 2.1 kcal per capsule. The placebo only consisted of refined corn oil, gelatin, glycerin, and purified water, with a caloric content of 3.1 kcal per capsule. The placebo and vitamin E treatments were identical in color, smell, appearance, packaging, and weight, making them indistinguishable to both researchers and participants. The double-blind allocation was facilitated by the interactive network response system (IWRS) using the dynamic randomization method.

Throughout the intervention, all participants received personalized lifestyle guidance guided by dieticians, which was detailed in Supplemental Methods. Daily food intake and exercise were recorded by a mobile phone application (Figure S1), and interim contact via telephone or WeChat was maintained monthly. Follow-up visits were conducted every 12 weeks. These contacts and visits were designed to promote retention, encourage compliance with the trial regimen, and monitor for adverse events.

Liver biopsies

Liver biopsies were performed at baseline and after 96 weeks of treatment. While initial eligibility assessments and tissue preparations were conducted locally by pathologists at each site, the final analysis was based on centrally prepared specimens for both baseline and post-treatment biopsies. Each biopsy was assessed by three independent hepatopathologists to determine NAS, including steatosis, lobular inflammation, hepatocyte ballooning, and fibrosis stage. Biopsies were read unpaired, and the central pathologists were blinded to treatment assignments as well as to any subject-specific clinical, laboratory, and imaging information. For cases with discordant assessments, a consensus was reached centrally before unblinding.

Outcomes

The primary endpoint was improvement in hepatic histology after 96 weeks of treatment. The definition of histologic improvement was based on the following criteria: either a reduction in NAS score of at least 2 points or a post-treatment NAS score of 3 points or less; a minimum 1-point improvement in score for ballooning or inflammation; and no worsening of fibrosis stage. A decrease in NAS score by at least 2 points was required for subjects with NAS of 2–4 points assessed by the central pathologists.

The exploratory secondary endpoint was an improvement in liver fibrosis by $\geq$ 1 stage (NASH CRN fibrosis score) and no worsening of steatohepatitis (defined as no increase in NAS for steatosis, inflammation, or ballooning). Other supportive secondary histologic endpoints included resolution of steatohepatitis on overall histopathological reading and no worsening of liver fibrosis; improvement in fibrosis by $\geq$ 1 stage or steatohepatitis resolution without fibrosis worsening; changes in histologic features from baseline to week 96 in total NAS; and changes of NAS components (steatosis, inflammation, or ballooning) and fibrosis score. Non-invasive secondary endpoints included changes from baseline to week 96 in serum biochemical levels, lipids, metabolic factors, cytokeratin 18 fragment M30/M65 (CK18 M30/M65), proinflammatory cytokines (TNF-α and IL-6), CAP, and LSM assessed by FibroScan.

Safety endpoints were assessed at each visit. Adverse events were graded for severity using Common Terminology Criteria for Adverse Events Version 4.0.

Laboratory testing

HP genotypes (HP 1-1, HP 2-1 and HP 2-2) have been reported to be associated with discrepant pharmacologic responses to vitamin E in participants with MASH.[27] Based on our previous study, the HP2 allele is more common than the HP1 allele in the Chinese population.[28] Therefore, Hp genotyping polymorphism was determined for all participants as detailed in our previous study.[28]

During follow-up visits, participants underwent physical examinations, vital sign measurements, FibroScan evaluations, and serum biochemical markers, lipids, metabolic indicators, and cytokines assessments.[19] Gas- and liquid-chromatography with mass spectrometry were performed to test vitamin E levels (Figure S4). Adverse events, observed by the investigator or reported by participants, were recorded and evaluated for severity, duration, outcome, and possible relationship to the study treatment. BNP and PSA (for male participants) were measured before and after treatment.

Recommendations for diet and exercise

Diet recommendations: (1) Advocate a varied and balanced diet with controlled total caloric intake. Avoid overly refined foods, and incorporate both whole and refined grains as staple foods; (2) Carbohydrates should make up about 55% of total energy intake, ideally from whole grains such as oats, buckwheat, brown rice, and millet. Avoid refined sugars, honey, fruit juice, jams, preserves, and other sweets, and reduce consumption of sucrose-containing beverages; (3) Fat should comprise less than 25% of total caloric

intake, with a focus on reducing saturated and trans fats. Vegetable oils, such as soybean oil, salad oil, and moderate amounts of tea oil or olive oil, are recommended to support healthy fat metabolism. Avoid animal fats (except fish oil) and limit foods high in cholesterol, such as animal organ meats, egg yolks, squid, sardines, bone marrow, and fish roe, keeping cholesterol intake below 300 mg per day; (4) Protein intake should be approximately 1.5 g per kg of body weight, with an emphasis on high-quality protein sources. Options include skim milk, egg whites, fish, lean meats, and soy products; (5) Increase dietary fiber to at least 24 g per day by consuming plenty of fresh vegetables and fruits, which provide essential vitamins and minerals for normal body metabolism. Dietary fiber can help reduce cholesterol formation, limit fat and sugar absorption, and thus aid in lowering blood lipids and plasma glucose levels; (6) Avoid alcohol and limit spicy or strongly flavored foods. Opt for a light diet with daily salt intake below 5 g; (7) Use cooking methods like steaming, boiling, braising, stewing, decocting, and simmering, while avoiding deep-frying or stir-frying.

Exercise recommendations: (1) Engage in moderate to low-intensity aerobic exercises, such as walking, cycling, or jogging, at least four times per week, totaling a minimum of 150 min per week; (2) During the initial phase, start with slightly lower intensity and shorter duration to avoid joint and muscle strain, then gradually increase over time. Each session should include a 5–10 min warm-up and at least 5 min of cool-down activities; (3) If any discomfort arises during exercise, such as significant fluctuations in blood glucose, marked fatigue, or difficulty in recovery, patients should reduce the exercise intensity or stop immediately; (4) Stop exercise immediately if symptoms like shortness of breath, pale complexion, nausea, or vomiting occur. Take appropriate measures if necessary; (5) Follow the principles of gradual progression, long-term consistency, and safety.

QUANTIFICATION AND STATISTICAL ANALYSES

According to the PIVENS study,[7] the sample size was calculated based on the assumption of an approximately 0.43 improvement rate in the vitamin E group and 0.19 in the placebo group with an α level of two-sided type I error of 0.05 and a β level of 0.2. Factoring in an estimated dropout rate of 10%, the sample size was finally calculated as 124 participants; a total of 124 participants were estimated to enroll for primary endpoint evaluation, with a power value of 0.85.

Quantitative variables were expressed as mean $\pm$ SD and compared via the Student's t test, and a QQ plot was applied for the normality test. Binary variables were presented as number (proportion). The analysis of the primary endpoint and exploratory secondary endpoints was performed in the mITT population. Binary outcomes were analyzed by a generalized linear model, generating OR and 95% CI as shown in Figure 2, Table S1 and S2. Chi-square test was applied for analyzing changes in each component of histologic features in Table S3. Analysis of each component of NAS and fibrosis scores were performed with the WR method to estimate between-group differences in Figure 3.[29] WR is a non-parametric approach that does not require any normality assumption for continuous outcome variables. The WR was determined as the total number of winners (successful outcomes favoring the treatment) divided by the total numbers of losers (successful outcomes favoring the control) among all pairwise comparisons between treatment and placebo groups. An estimated WR greater than 1 indicates that a treatment effect is in favor of vitamin E, while a WR less than 1 means a treatment effect favoring placebo. A WR equal to 1 suggests no difference between the groups. The 95% CI for the WR was obtained by asymptotic theory.[30] Participants with missing histology data were classified as non-responders. For secondary continuous endpoints, an analysis of covariance (ANCOVA) model was used shown in Figure 4, Table 2, and Table S4, with the absolute change from baseline as the dependent variable, treatment group as a fixed factor, and baseline value as a covariate. Sensitivity analysis was performed based on histology assessment with local readings at baseline and central reading after 96-week treatment and in participants with MASH F1$\sim$F3. All statistical analyses were performed with SAS 9.4, R version 4.2.3, and Prism 10.1.2.

ADDITIONAL RESOURCES

The study was registered at clinicaltrials.gov (NCT02962297).

Cell Reports Medicine, Volume *6*

Supplemental information

Vitamin E (300 mg) in the treatment of MASH:

A multi-center, randomized, double-blind,

placebo-controlled study

Yu Song, Wenjing Ni, Minghua Zheng, Huiping Sheng, Jing Wang, Shilong Xie, YongFeng Yang, Xiaoling Chi, Jinjun Chen, Fangping He, Xiaotang Fan, Yuqiang Mi, Jing Zhang, Bingyuan Wang, Lang Bai, Wen Xie, Bihui Zhong, Yee Hui Yeo, Fajuan Rui, Shufei Zang, Jie Li, Junping Shi, and the Chinese NAFLD Clinical Research Network (CNAFLD CRN)

Supplementary Information

Table of Content

Supplementary Figures

Figure S1. Information collection of diet and exercise. Related to STAR Methods.

Figure S2. Typical picture with improvements in histology. Related to Figure 2.

Figure S3. Safe characteristics of the study subjects (Safety set). Related to Table 3.

Figure S4. Blood concentration of vitamin E. Related to STAR Methods.

Supplementary Tables

Table S1 Sensitivity analyses (mITT). Related to Figure 2.

Table S2 Subgroup analyses. Related to Figure 2.

Table S3 Changes in other histologic features in mITT. Related to Figure 3.

Table S4. Change between baseline and week 96 in secondary endpoints and after 120 weeks (96 weeks of treatment followed by 24 weeks off-treatment). Related to Table 2.

Table S5 Combination therapy. Related to Table 3.

Figure S1. Information collection of diet and exercise. Related to STAR Methods.

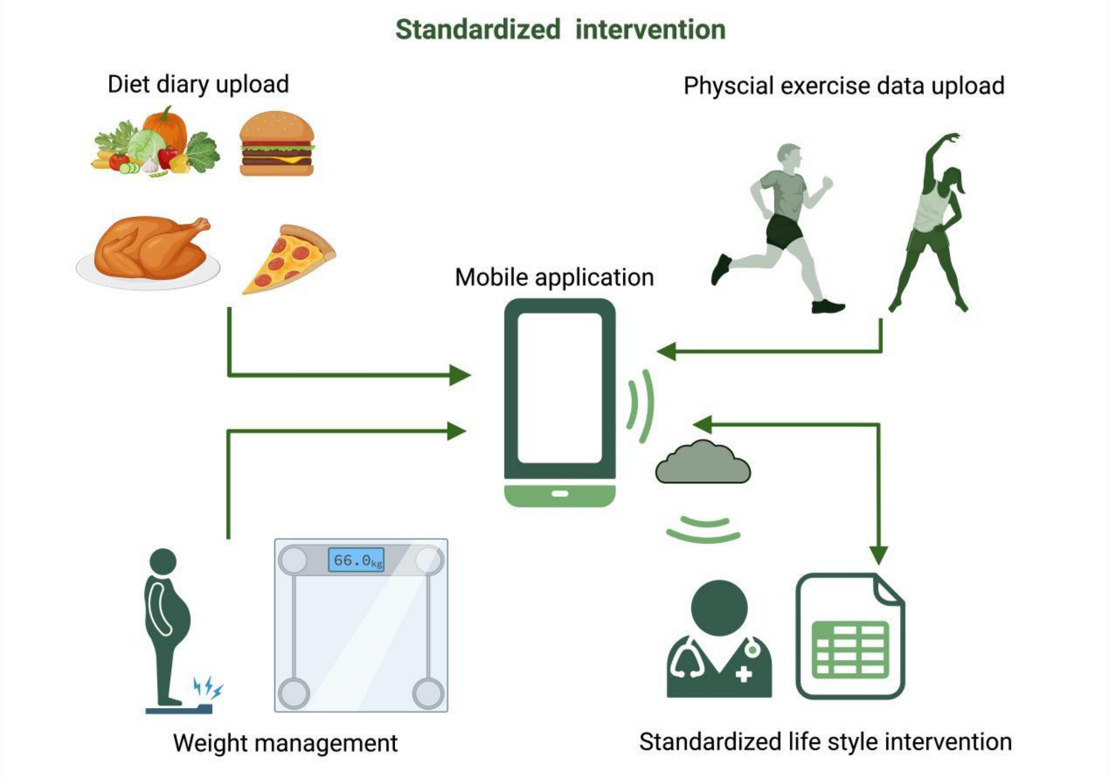

All participants were given personalized lifestyle advice by dieticians. Every patient's daily food consumption and exercise were collected using a mobile phone application.

Figure S2. Typical picture with improvements in histology. Related to Figure 2.

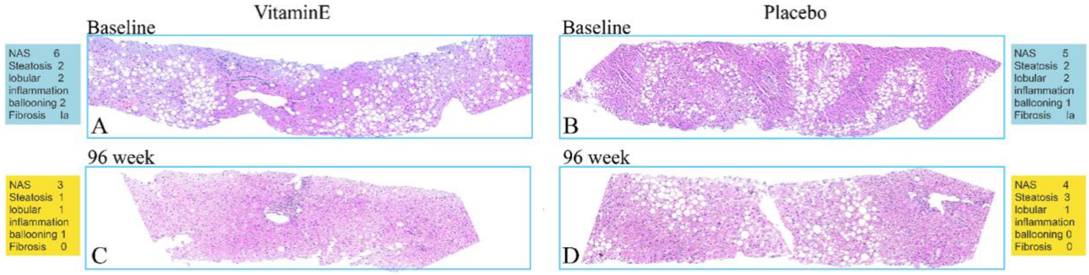

Micrographs of a patient with marked histological improvement after 96 weeks of treatment. Panel A (Vitamin E group H&E) and B (Placebo group H&E) from the pre-treatment biopsy showed the classic features of steatohepatitis. Panel C (Vitamin E group H&E) and D (Placebo group H&E) from the post-treatment biopsy showed the improvement in all features of steatohepatitis. Abbreviations: H&E: hematoxylin-eosin.

Figure S3. Safe characteristics of the study subjects (Safety set). Related to Table 3.

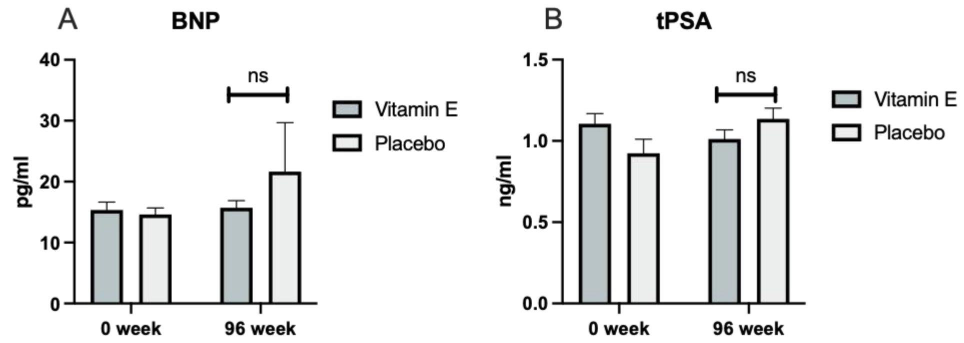

(A): BNP levels are assessed in each group within the safety set population (n=58 in the vitamin E group; n=66 in the placebo group); (B): tPSA levels are assessed in males of each group within the safety set population (n=42 in the vitamin E group; n=50 in the placebo group). Data are presented as mean in each group. Error bars represent standard deviations. Abbreviations: BNP, brain natriuretic peptide; tPSA, total prostate specific antigen.

Figure S4. Blood concentration of vitamin E. Related to STAR Methods.

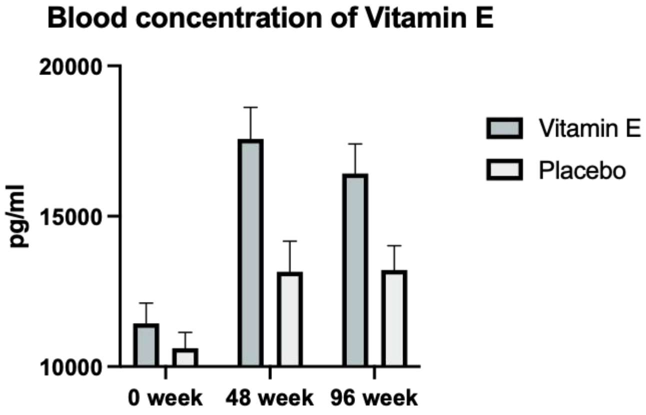

Data are presented as mean in each group. In each time point, the number in the vitamin E and the placebo groups are 39 and 51, respectively. Error bars represent standard deviations.

Table S1. Sensitivity analyses (mITT). Related to Figure 2.

	Vitamin E 300mg Group (n=58)	Placebo group (n=64)	Odds ratio (95% CI)	*P* value
Exclude if missed or outside time window	17 (43.6)	9 (17.6)	3.6 (1.3, 10.7)	0.01
Local reading at baseline, central reading at week 96				
Exclude if missing or outside time window	20 (51.3)	15 (29.4)	1.7 (1.0, 2.9)	0.04
Exploratory secondary endpoints				
Fibrosis improvement	15 (25.9)	10 (15.6)	1.7 (0.8, 3.4)	0.16
Subjects with improvement (%)	25.7%	15.7%		
Sensitivity analyses				
Exclude if missed or outside time window	13 (33.3)	9 (17.6)	2.0 (1.0, 3.9)	0.09
Among subjects F1-3	15 (50.0)	10 (25.6)	2.0 (1.0, 3.7)	0.04
Local reading at baseline, central reading at week 96				
Exclude if missing or outside time window	17 (43.6)	10 (19.6)	2.2 (1.1, 4.3)	0.01
Among subjects F1-3	17 (35.4)	10 (17.2)	2.1 (1.0, 4.1)	0.03

Data are presented with mean (standard deviation).

Table S2. Subgroup analyses for primary outcome (mITT). Related to Figure 2.

	Vitamin E 300mg group (n = 58)	Placebo group (n = 64)	Odds ratio (95% CI)	*P* value	*P* for Interaction
Hp genotype					
Hp 2-1	1 (100.0%)	0 (0.0%)	NA	0.08	0.99
Hp 2-2	16 (28.1%)	9 (14.5%)	(0.8, 6.5)	0.07	
Gender					
Male	14 (33.3%)	5 (10.2%)	4.4 (1.3, 17.1)	0.01	0.06
Female	3 (18.8%)	4 (26.7%)	0.6 (0.08, 4.7)	0.60	
Age at baseline					
< 40	10 (30.3%)	3 (8.1%)	4.9 (1.1, 30.2)	0.02	0.18
≥40	7 (28.0%)	6 (22.2%)	1.4 (0.3, 5.9)	0.63	
BMI					
< 25	4 (22.2%)	5(14.7%)	1.7 (0.3, 9.0)	0.50	0.52
≥25	13 (32.5%)	4 (13.3%)	3.1 (0.8, 14.7)	0.06	
NAS					
2-4	3 (13.0%)	4 (14.3%)	0.9 (0.1, 6.0)	0.90	0.13
5-8	14 (40.0%)	5 (13.9%)	4.1 (1.2, 16.6)	0.01	

Data are presented with n (%). Abbreviations: CI, confidence interval; NAS, Non-alcoholic fatty liver disease score; BMI, body mass index; NA, not applicable.

Table S3. Changes in other histologic features (mITT). Related to Figure 3.

	Vitamin E 300mg group (n = 58)	Placebo group (n = 64)	*P* value
Steatosis			0.13
n (%) worse	2 (3.5%)	8 (12.5%)	
n (%) same	32 (55.2%)	37 (57.8%)	
n (%) better	24 (41.4%)	19 (29.7%)	
Lobular inflammation			0.07
n (%) worse	2 (3.5%)	10 (15.6%)	
n (%) same	35 (60.3%)	31 (48.4%)	
n (%) better	21 (36.2%)	23 (35.9%)	
Hepatocellular ballooning			0.60
n (%) worse	8 (13.8%)	7 (10.9%)	
n (%) same	40 (69.0%)	41 (64.1%)	
n (%) better	10 (17.2%)	16 (25.0%)	
Total NAFLD Activity Score			0.29
n (%) worse	5 (8.6%)	12 (18.8%)	
n (%) same	27 (46.6%)	26 (40.6%)	
n (%) better	26 (44.8%)	26 (40.6%)	
Fibrosis			0.09
n (%) worse	3 (5.2%)	11 (17.2%)	
n (%) same	38 (65.5%)	39 (60.9%)	
n (%) better	17 (29.3%)	14 (21.9%)	

Data are presented with n (%).

Table S4. Change between baseline and week 120 in secondary endpoints (mITT). Related to Table 2.

	Vitamin E 300 mg group (n = 58)	Placebo group (n = 64)	Point estimate (95% CI)	*P*-Value
**Changes in anthropometric parameters**				
BMI, kg/m^2	-0.1 (3.4)	0.1 (1.8)	0.5 (-0.3, 1.3)	0.24
WHR*	0.0 (0.1)	0.0 (0.1)	0.0 (0.0, 0.0)	0.60
**Liver enzyme**				
ALT, U/L	-10.1 (44. 8)	-11.5 (39. 8)	1.5 (-11.2, 14.2)	0.81
AST, U/L	-4.1 (18.6)	-3.4 (17.7)	-2.3 (-7.5, 2.8)	0.38
γ-GT, U/L	0.2 (31.7)	-7.3 (30.3)	6.9 (2.8, 16.6)	0.16
AKP, U/L	-1.1 (31.6)	-4.6 (18.7)	4.1 (-3.8, 12.0)	0.31
TBIL, μmol/L	-1.5 (9.7)	-0.7 (7.7)	-2.0 (-4.0, 0.2)	0.07
**Lipids**				
TG, mmol/L	0.3 (1.2)	0.4 (1.4)	-0.1 (-0.6, 0.3)	0.56
TC, mmol/L	0.4 (1.1)	0.3 (0.9)	0.2 (-0.2, 0.5)	0.28
HDL*, mmol/L	0.1 (0.2)	0.0 (0.2)	0.1 (0.0, 0.2)	0.01
LDL, mmol/L	0.5 (1.1)	0.1 (0.8)	0.4 (0.1, 0.7)	0.01
**Metabolic factors**				
Fasting serum glucose*, mmol/L	0.1 (1.4)	0.3 (1.4)	-0.4 (-0.9, 0.2)	0.17
Fasting insulin, pmol/L	19.2 (104.0)	3.9 (87.0)	15.4 (-13.3, 44.1)	0.29
HOMA-IR	6.8 (34.2)	1.5 (21.1)	5.3 (-3.8, 14.5)	0.25
2h blood glucose, mmol/L	-0.2 (2.0)	0.4 (2.5)	-0.7 (-1.4, 0.0)	0.05
**FibroScan Assessment**				
Controlled attenuation parameter, dB/m	3.0 (66.6)	6.0 (54.2)	-2.8 (-18.2, 12.7)	0.72
Liver stiffness measuremen, kPa	-1.4 (3.3)	1.0 (6.4)	-1.5 (-3.2, 0.2)	0.09

Data are presented with mean (SD). An analysis of covariance (ANCOVA) model was used, with the absolute change from baseline as the dependent variable, treatment group as a fixed factor, and baseline value as a covariate.

* Both baseline BMI and corresponding baseline value were included as covariates in ANCOVA model considering the *P*-value for the main effect of baseline BMI was less than 0.05.

Abbreviations: BMI, body mass index, calculated as weight in kilograms divided by height in meters squared; WHR, waist-to-hip ratio, calculated as waist circumference in meters divided by hip circumference in meters; ALT, alanine aminotransferase; AST, aspartate aminotransferase; γ-GT, Glutamyl peptidyl transferase; AKP, alkaline phosphatase; TG, total triglycerides; TC, total cholesterol; LDL, low-density lipoprotein; HDL, high-density lipoprotein; FPG, fasting plasma glucose; FINS, fasting insulin; HOMA-IR, Homeostasis Model Assessment for Insulin Resistance, calculated as insulin × glucose/22.5, where the unit of measure for insulin is μIU/mL and the unit of measure for glucose is mmol/L; CAP, controlled attenuation parameter; LSM, liver stiffness measurement.

Table S5. Combination therapy (FAS). Related to Table 3.

	Vitamin E (n=58)		Placebo (n=66)	
	n	%	n	%
Cardiovascular system	14	24.1	8	12.5
Calcium channel blockers	5	8.6	3	4.7
Amlodipine	2	3.5	2	3.1
Levoamlodipine Maleate	2	3.5	0	0.0
Felodipine	0	0.00	1	1.6
Nifedipine	1	1.7	0	0.0
Drugs that act on the renin-angiotensin system	4	6.9	4	6.3
Valsartan	0	0.0	3	4.7
Indapamide; Perindopril tert-butylamine	0	0.0	1	1.6
Hydrochlorothiazide; Irbesartan	1	1.7	0	0.0
Hydrochlorothiazide; Valsartan	1	1.7	0	0.0
Telmisartan	1	1.7	0	0.0
Benazepril Hydrochloride	1	1.7	0	0.0
Lipid Regulatory Drugs	1	1.7	2	3.1
Atorvastatin Calcium	1	1.7	1	1.6
Pitavastatin calcium	0	0.0	1	1.6
Rosuvastatin Calcium	0	0.0	1	1.6

Data are presented with n (%).
